# Dual Specificity Phosphatase 1 Regulates Human Inducible Nitric Oxide Synthase Expression by p38 MAP Kinase

**DOI:** 10.1155/2011/127587

**Published:** 2011-04-19

**Authors:** Tuija Turpeinen, Riina Nieminen, Ville Taimi, Taina Heittola, Outi Sareila, Andrew R. Clark, Eeva Moilanen, Riku Korhonen

**Affiliations:** ^1^The Immunopharmacology Research Group, University of Tampere School of Medicine and Tampere University Hospital, Medisiinarinkatu 3, 33014 Tampere, Finland; ^2^The Kennedy Institute of Rheumatology, Imperial College London, London W6 8LH, UK

## Abstract

The role of dual specificity phosphatase 1 (DUSP1) in inducible nitric oxide synthase (iNOS) expression in A549 human pulmonary epithelial cells, J774 mouse macrophages and primary mouse bone marrow-derived macrophages (BMMs) was investigated. iNOS expression was induced by a cytokine mixture (TNF, IFN*γ* and IL-1*β*) in A549 cells and by LPS in J774 cells, and it was inhibited by p38 MAPK inhibitors SB202190 and BIRB 796. Stimulation with cytokine mixture or LPS enhanced also DUSP1 expression. Down-regulation of DUSP1 by siRNA increased p38 MAPK phosphorylation and iNOS expression in A549 and J774 cells. In addition, LPS-induced iNOS expression was enhanced in BMMs from DUSP1^(−/−)^ mice as compared to that in BMMs from wild-type mice. The results indicate that DUSP1 suppresses iNOS expression by limiting p38 MAPK activity in human and mouse cells. Compounds that enhance DUSP1 expression or modulate its function may be beneficial in diseases complicated with increased iNOS-mediated NO production.

## 1. Introduction

Nitric oxide (NO) is a gaseous signaling molecule that regulates various physiological and pathophysiological processes in many tissues and organ systems. NO is synthesized from L-arginine in a reaction catalyzed by nitric oxide synthase (NOS) enzyme. Three NOS enzyme isoforms exist: neuronal NOS (nNOS), inducible NOS (iNOS), and endothelial NOS (eNOS). nNOS and eNOS are constitutively expressed, and, in general, they produce relatively small amounts of NO in the context of physiological regulation of cellular and tissue functions. The expression of iNOS is induced by a number inflammatory and other stimuli, such as inflammatory cytokines, bacterial products, and hypoxia. NO is an important effector molecule in microbicidal host defense, and it serves as a regulatory and proinflammatory molecule in acute and chronic inflammatory responses [1–4]. 

The expression of iNOS is regulated at transcriptional and posttranscriptional levels. There are considerable differences in the transcriptional regulation of mouse and human iNOS expression. *Mouse* iNOS promoter activity is substantially induced by interferon (IFN)*γ* and bacteria-derived substances, such as lipopolysaccharide (LPS). iNOS promoter contains two regions responsive to LPS and IFNs. The proximal region is located between −48 and −209 bp upstream of transcriptional start site and contains binding site for nuclear factor *κ*B (NK-*κ*B) and is essential for NF-*κ*B-dependent inducible iNOS promoter activity. The distal region, at position −913 to −1029 bp, contains NF-kB binding site, gamma-activated site (GAS) and two copies of interferon-stimulated response element (ISRE) [5]. Interferon-stimulated gene factor 3 (ISGF3; a heterotrimer of signal transducer and activator of transcription (STAT)1, STAT2, and interferon regulatory factor (IRF)9) bound to the distal responsive element and NF-*κ*B bound to the proximal responsive element have been shown to cooperate to induce iNOS expression [6]. Several other transcription factors have been shown to regulate mouse iNOS transcription including IRF-1, Octamer factor (Oct-1), activating protein-1 (AP-1), and high-mobility group protein HMG-I(Y) [2, 7]. 

Transcriptional regulation of *human* iNOS expression shows complexity. Human iNOS promoter shows basal promoter activity, and regulatory elements involved in the cytokine-induced human iNOS transcription are located between −3.8 and −16 kb upstream of the transcriptional start site [7, 8]. A number of transcription factors contribute to human iNOS transcription. NF-*κ*B and STAT1 are considered to be the key transcription factors regulating human iNOS transcription [9, 10]. AP-1 has been reported to have positive and negative effects on human iNOS promoter activity [11, 12]. Several other transcription factors have been shown to be involved in human iNOS transcription including Oct-1, cAMP-responsive element-binding protein, CCAAT-enhancer box-binding protein, STAT3, NF-IL6, and hypoxia-induced factor-1 [7]. 

Mitogen-activated protein kinases (MAPKs) have been shown to regulate iNOS expression, especially by posttranscriptional mechanisms. iNOS mRNA stability has been shown to be regulated by p38 MAPK and Jun N-terminal kinase (JNK) [13–15]. Other factors involved in the regulation of iNOS expression at posttranscriptional level include transforming growth factor *β*, glucocorticoids, and inhibitors of calcineurin [16–18]. Proteins that bind to the 3′ untranslated region of iNOS mRNA and regulate iNOS expression at posttranscriptional level include embryonic lethal abnormal visual RNA-binding protein HuR, tristetraprolin, KH-type splicing regulatory protein, and heterogeneous nuclear ribonucleoprotein D and I [13, 19–22]. 

MAPKs are a group of serine/threonine protein kinases involved in the cellular signal transduction, and the members of this signalling pathway group include p38 MAPK, JNK and p42/44 ERK. They are activated via phosphorylation of specific tyrosine and threonine residues by the upstream kinases. MAPKs regulate various physiological processes, including cell growth, differentiation, and stress responses, and p38 and JNK are associated with the regulation of inflammatory and immune responses [23–25]. There are four p38 MAPK isoforms (p38*α*, p38*β*, p38*γ*, and p38*δ*), all encoded by separate genes. Especially p38*α* and p38*β* have been found to regulate immune response [24–26]. Many different stimuli, including LPS, cytokines and growth factors, activate p38 MAPK pathway [27–31]. The activation of p38 MAPK is involved in the expression of several inflammatory genes, such as tumor necrosis factor (TNF), interleukin(IL)-1, IL-6, IL-8, cyclooxygenase-2 (COX-2) and iNOS [13, 26, 27, 32–35]. p38 MAPK inhibitors have been shown to suppress the expression of inflammatory cytokines, progression of arthritis, and pulmonary fibrosis in animal models and attenuate inflammatory response during endotoxemia in humans [36–38].

Dual specificity phosphatases (DUSPs) are a group of protein phosphatases that dephosphorylate phosphotyrosine and phosphoserine/threonine residues in their target proteins and regulate several intracellular signaling pathways. DUSPs associated with MAPK pathways (at least ten members) differ from each other by substrate specificity, tissue distribution, cellular localization, and expressional pattern [39, 40]. DUSP1 dephosphorylates tyrosine and threonine residues in MAPK Thr-Xaa-Tyr activation motif and thereby inactivates MAPK. DUSP1 has substrate specificity towards p38 and JNK over ERK [41–44]. DUSP1 deficient mice produce elevated levels of inflammatory cytokines and develop more severe NO-mediated hypotensive response and organ failure after administration of LPS or peptidoglycan and lipoteichoic acid [41, 43, 45, 46]. 

We have previously reported that DUSP1 negatively regulates IL-6, IL-8 and COX-2 expression in A549 human epithelial cells [47]. In addition, we have recently shown that the suppression of the expression of COX-2, matrix metalloproteinase 3 (MMP-3), and IL-6 by antirheumatic drug aurothiomalate in mouse and human chondrocytes and cartilage is mediated by DUSP1 [48]. In the present study, we investigated the effect of DUSP1 on the expression of iNOS in human and murine cells. The main finding was that DUSP1 suppresses iNOS expression by limiting p38 signaling in human cells, which is a novel finding, and this was observed in mouse macrophages also. 

## 2. Materials and Methods

### 2.1. Materials

Reagents were obtained as follows. BIRB 796 (1-(5-tertbutyl-2-p-tolyl-2H-pyrazol-3-yl)-3(4-(2-morpholin-4-yl-ethoxy)naphthalen-1-yl)urea, Axon MedChem, Groningen, The Netherlands), SB202190 (4-[4-(4-Fluorophenyl)-5-(4-pyridinyl)-1H-imidazol-2-yl] phenol, Tocris Bioscience, Bristol, UK), recombinant human TNF, recombinant human IFN*γ*, recombinant human IL-1*β*, recombinant mouse macrophage colony-stimulating factor (M-CSF) (R&D Systems Inc., Minneapolis, Mass, USA), medetomidine (Domitor 1 mg/mL, Orion Oyj, Espoo, Finland), and ketamine (Ketalar 10 mg/mL, Pfizer Oy Animal Health, Helsinki, Finland) were obtained as indicated. All other reagents were purchased from Sigma Chemicals Co. (Saint Louis, Mo, USA) unless otherwise stated below.

### 2.2. Cell Culture

A549 human lung epithelial cells (ATCC, Manassas, Va, USA) were cultured at 37°C in 5% CO_2_ atmosphere in Ham's F12K (Kaighn's modification) medium supplemented with 5% heat-inactivated fetal bovine serum (FBS), 100 U/mL penicillin, 100 *μ*g/mL streptomycin, and 250 ng/mL amphotericin B (all from Invitrogen, Paisley, UK). J774 macrophages (ATCC, Manassas, Va, USA) were cultured at 37°C in 5% CO_2_ atmosphere in Dulbecco's modified Eagle's medium with Ultraglutamine 1 (Lonza, Verviers Sprl, Verviers, Belgium) supplemented with 5% heat-inactivated FBS, 100 U/ml penicillin, 100 *μ*g/mL streptomycin, and 250 ng/mL amphotericin B. 

For experiments, A549 cells (4 × 10^5^ cells/well) were seeded on a 24-well plate and grown for 48 h prior to the experiments. J774 cells (2 × 10^5^ cells/well) were seeded on a 24-well plate and grown for 72 h prior to the experiments. BIRB 796 and SB202190 were dissolved in DMSO. BIRB 796, SB202190 at concentrations indicated, or DMSO (v/v 0.1%) were added to the cells in fresh culture medium containing 5% FBS and antibiotics 30 min prior to the stimulation with a cytokine mixture containing TNF, IFN*γ*, and IL-1*β* (10 ng/mL each) or LPS (10 ng/mL). Cells were further incubated for the time indicated.

### 2.3. Animals and Isolation and Culture of Bone Marrow Macrophages

Murine bone marrow macrophages (BMMs) were obtained from wild-type and DUSP1^(−/−)^ C57BL/6 mice. Inbred C57BL/6 DUSP1^(−/−)^ mice were originally generated by the R. Bravo laboratory at Bristol-Myers Squibb Pharmaceutical Research Institute [49], and the wild-type mice originated from the same strain. The study was approved by the Animal Care and Use Committee of the University of Tampere and the respective provincial committee for animal experiments. Female mice aged 10–12 weeks were used in the study. The mice were anesthetized by intraperitoneal injection of  0.05 mg/100 g body weight of medetomidine and 7.5 mg/100 g body weight of ketamine. Finally, mice were euthanized by cervical dislocation. Bone marrow cells were obtained by aspiration with sterile syringe needle from femur and fibia. BMMs were generated from bone marrow cells with 5–7 days of incubation in RPMI 1640 medium supplemented with 10% heat-inactivated fetal calf serum (FCS), 100 U/mL penicillin, 100 *μ*g/mL streptomycin and 10 ng/mL M-CSF. BMMs (1 × 10^6^ cells/well) were then seeded on a 24-well plate and cultured overnight in complete culture medium. BMMs were then serum-starved overnight. In the beginning of the experiment, LPS was added to the cells along with the culture medium containing 10% FCS and antibiotics, and BMMs were incubated for the time indicated.

### 2.4. Preparation of Cell Lysates for Western Blot Analysis

At the indicated time points, culture medium was removed. Cells were rapidly washed with ice cold PBS and solubilized in cold lysis buffer containing 10 mM Tris-HCl, 5 mM EDTA, 50 mM NaCl, 1% Triton-X-100, 0.5 mM phenylmethylsulfonyl fluoride, 1 mM sodiumorthovanadate, 20 *μ*g/mL leupeptin, 50 *μ*g/mL aprotinin, 5 mM sodium fluoride, 2 mM sodium pyrophosphate, and 10 *μ*M *n*-octyl-D-glucopyranoside. After incubation for 20 min on ice, lysates were centrifuged (12 000 g, 10 min) and supernatants were collected, mixed in a ratio of 1: 4 with SDS loading buffer (62.5 mM Tris-HCl, pH 6.8, 10% glycerol, 2% SDS, 0,025% bromophenol blue, and 5% mercaptoethanol) and stored at −20°C until analyzed. Protein concentrations in the samples were measured by the Coomassie blue method.

### 2.5. Western Blotting

Actin (sc-1616-R), DUSP1 (M-18, sc-1102), lamin A/C (sc-20681), and polyclonal antirabbit (sc-2004) and polyclonal antigoat (sc-2020) antibodies were obtained from Santa Cruz Biotechnology (Santa Cruz, Calif, USA). Phospho-p38 MAPK (Cat. no. 9218), p38 MAPK (Cat. no. 9212), mitogen-activated protein kinase-activated protein kinase 2 (MK2) (Cat. no. 3042) and phospho-MK2 (27B7, Cat. no. 3007) antibodies (Cell Signaling Technology Inc, Beverly, Mass, USA) were obtained as indicated. Prior to Western blot analysis, the protein samples were boiled for 10 min. Equal aliquots of protein (10–20 *μ*g) were loaded on a 10% SDS-polyacrylamide electrophoresis gel and separated by electrophoresis. Proteins were transferred to Hybond enhanced chemiluminescence nitrocellulose membrane (Amersham, Buckinghamshire, UK) by semidry electroblotting. After transfer, the membrane was blocked in TBS/T (20 mM Tris-base pH 7.6, 150 mM NaCl, and 0.1% Tween-20) containing 5% nonfat milk for 1 h at room temperature. For detection of phospho-proteins, membranes were blocked in TBS/T containing 5% bovine serum albumin. Membranes were incubated overnight at 4°C with the primary antibody and for 1 h with the secondary antibody in room temperature, and the chemiluminescent signal was detected by ImageQuant LAS 4000 mini (GE Healthcare Bio-Sciences AB, Uppsala, Sweden). The chemiluminescent signal was quantified with ImageQuant TL 7.0 Image Analysis Software.

### 2.6. NO Measurement

Cells were incubated with compounds of interest for 24 h. Culture medium was then collected, and nitrite (a stable metabolite of NO in aqueous conditions) levels were measured by the Griess reaction.

### 2.7. RNA Extraction and Quantitative RT-PCR

Primers and probes for quantitative RT-PCR were obtained from Metabion International AG (Martinsried, Germany). At the indicated time points, culture medium was removed and total RNA extraction was carried out with GenElute Mammalian Total RNA Miniprep Kit (Sigma-Aldrich, St Louis, Mo, USA) according to the manufacturer's instructions. Total RNA was reverse-transcribed to cDNA using TaqMan Reverse Transcription reagents and random hexamers (Applied Biosystems, Foster City, Calif, USA). cDNA obtained from the RT-reaction was diluted 1: 20 with RNAse-free water and was subjected to quantitative PCR using TaqMan Universal PCR Master Mix and ABI PRISM 7000 Sequence detection system (Applied Biosystems, Foster City, Calif, USA). The primer and probe sequences and concentrations (Table 1) were optimized according to manufacturer's guidelines in TaqMan Universal PCR Master Mix Protocol part number 4304449 revision C. Expression of human Lamin A/C mRNA and human DUSP1 mRNA were measured using TagMan Gene Expression Assays (Applied Biosystems, Foster City, Calif, USA). PCR reaction parameters were as follows: incubation at 50°C for 2 min, incubation at 95°C for 10 min, and thereafter 40 cycles of denaturation at 95°C for 15 s and annealing and extension at 60°C for 1 min. Each sample was determined in duplicate. A standard curve method was used to determine the relative mRNA levels as described in the Applied Biosystems User Bulletin: A standard curve for each gene was created using RNA isolated from A549 cells stimulated with cytokines (TNF, IL-1*β*, and IFN*γ*; 10 ng/mL each) and J774 cells stimulated with LPS (10 ng/mL). Isolated RNA was reverse-transcribed, and dilution series of cDNA ranging from 1 pg to 10 ng were subjected to real-time PCR. The obtained threshold cycle values were plotted against the dilution factor to create a standard curve. Relative mRNA levels in test samples were then calculated from the standard curve.

### 2.8. Downregulation of DUSP1 by siRNA

Human DUSP1 siRNA 1 (Cat. no. J-003484-09-0005) and human DUSP1 siRNA 2 (Cat. no. J-003484-10-0005) were purchased from Dharmacon (Dharmacon, Lafayette, Colo, USA). Lamin A/C siRNA (Cat. no. 1022050,) and nontargeting control siRNA (Cat. no. 1022076) were purchased from QIAGEN (QIAGEN, Valencia, Calif, USA). Mouse DUSP1 was silenced using ON-TARGET SMART pool (Dharmacon, Cat. no. L-040753-00-0005). siCONTROL nontargeting siRNA #1 (Dharmacon, Cat. no. D-001210-01) was used as a negative control siRNA in J774 cells. 

A549 cells were transfected with siRNA using HiPerFect transfection Reagent (QIAGEN) according to the manufacturer's instructions. Briefly, cells (1.25 × 10^5^ cells/well) were seeded on a 24-well plate in 500 *μ*L of medium with 5% FBS without antibiotics. For one well, 3 *μ*L of siRNA stock solution (2 *μ*M) was mixed with 1.5 *μ*L of transfection reagent in final volume of 100 *μ*L of medium, incubated for 5 min in room temperature, and applied over the cells. Cells were further incubated for 48 h. Fresh culture medium was changed and cytokines were added into the culture medium. Cells were further incubated for the time indicated, and gene expression was analyzed. 

J774 cells were transfected with siRNA using DharmaFECT 4 transfection reagent (Dharmacon, Lafayette, Colo, USA) according to the manufacturer's instructions. Briefly, cells (1 × 10^5^ cells/well) were seeded on a 24-well plate in 500 *μ*L of medium with 5% FBS without antibiotics and incubated overnight. For one well, the final transfection medium applied to the cells contained 25 *μ*L of siRNA stock solution (2 *μ*M) mixed with 1 *μ*L of transfection reagent in final volume of 500 *μ*L of medium. Cells were further incubated for 48 h. Fresh culture medium was changed, and LPS was added into the culture medium. Cells were further incubated for the time indicated, and gene expression was analyzed.

Transfection efficacy was monitored with green fluorescent siRNA oligos (siGLO green transfection indicator, Cat. no. D-001630-01, Dharmacon, Lafayette, Colo, USA) using Nikon Eclipse TS100 microscope (Nikon, Tokyo, Japan). Approximately 90% of the cells emitted green fluorescence signal when transfected with siGLO and HiPerFect (A549 cells) or siGLO and DharmaFECT 4 (J774 cells). Less than 5% of the cells emitted signal when cells were incubated siGLO oligos without transfection reagent.

### 2.9. Statistics

Results are expressed as the mean ± S.E.M. When appropriate, one-way ANOVA with Dunnett's or Bonferroni's post test was performed using GraphPad InStat version 3.05 for Windows 95/NT (GraphPad Software, San Diego, Calif, USA). Differences were considered significant at *P < .05, **P < .01, and ***P < .001.

## 3. Results

### 3.1. p38 MAPK Inhibitors SB202190 and BIRB 796 Downregulated iNOS Expression and NO Production in Response to Inflammatory Stimuli in A549 Cells and J774 Cells

**Figure 1:**
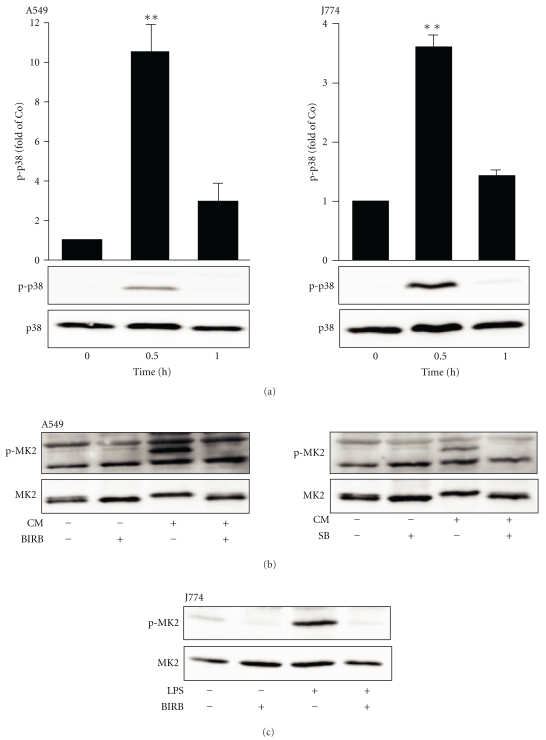
Phosphorylation of p38 MAPK and its substrate MK2 in response to stimulation with cytokine mixture or LPS in A549 and J774 cells. (a) A549 and J774 cells were stimulated with the cytokine mixture (CM: TNF, IFN*γ*, and IL-1*β*; 10 ng/mL each) or LPS (10 ng/mL), respectively for the time indicated. Cells were then harvested for protein extraction, and phosphorylation of p38 MAPK was detected by Western blot. The gel is a representative of six separate experiments with similar results. Chemiluminescent signal was quantified, and phosphorylated p38 MAPK was normalized against total p38 MAPK. Phosphorylation levels are expressed in arbitrary units, unstimulated cells set as 1, and the other values are related to that. Results are expressed as mean ± S.E.M.; *n* = 6. One-way ANOVA with Dunnett's posttest was performed, and statistical significance was indicated with **P < .01 compared with unstimulated cells. ((b) and (c)) The effect of SB202190 and BIRB 796 on the phosphorylation of MK2 in response to cytokine mixture in A549 and J774 cells. Cells were preincubated with SB202190 (1 *μ*M) or BIRB 796 (100 nM) for 30 min and stimulated with cytokine mixture (A549 cells) or LPS (J774 cells) for 30 min, and the phosphorylation of MK2 was detected by Western blot. The gels are representatives of six separate experiments with similar results.

A549 pulmonary epithelial cells and J774 macrophages were stimulated with a cytokine mixture (TNF, IFN*γ*, and IL-1*β*; 10 ng/mL each) and LPS (10 ng/mL), respectively, for the time indicated. Cells were then harvested for protein extraction, and the phosphorylation of p38 MAPK was detected by Western blot. p38 MAPK phosphorylation was increased in response to stimulation at time point of 30 min, and it was returned to the basal level in 1 h (Figure 1(a)). p38 MAPK inhibitors SB202190 and BIRB 796 have been reported to effectively inhibit p38 MAPK at 1 *μ*M and 100 nM concentrations, respectively [50]. To confirm the inhibiting effect of SB202190 and BIRB 796 on p38 MAPK activity in the current experimental condition, their effect on the phosphorylation of p38 MAPK substrate MK2 (47 kDa) was investigated in A549 cells and J774 cells. Cells were preincubated with p38 MAPK inhibitors SB202190 or BIRB 796 for 30 min and stimulated with the cytokine mixture or LPS for 30 min. The phosphorylation of MK2 was detected by Western blot. SB202190 and BIRB 796 inhibited MK2 phosphorylation at concentrations of 1 *μ*M and 100 nM, respectively (Figures 1(b) and 1(c)). These results confirmed that SB202190 and BIRB 796, at concentrations used, inhibited p38 MAPK function. 

**Figure 2:**
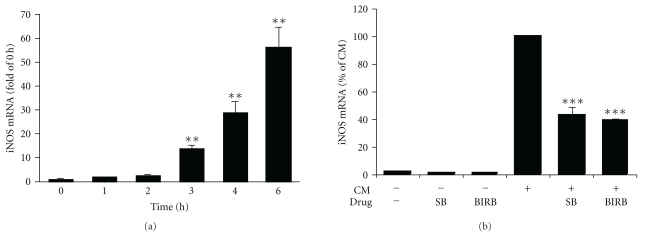
Expression of iNOS mRNA over time and effects of SB202190 and BIRB 796 on the expression of iNOS mRNA in response to cytokine mixture in A549 cells. (a) Cells were stimulated with cytokine mixture (CM: TNF, IFN*γ*, and IL-1*β*; 10 ng/mL each) for the time indicated, and total RNA was extracted. The expression of iNOS mRNA was determined by quantitative real-time RT-PCR, and mRNA expression was normalized against GAPDH mRNA. Unstimulated cells (0 h) were set as 1, and other values were related to that. Results are expressed as mean ± S.E.M., *n* = 3. One-way ANOVA with Dunnett's posttest was performed, and statistical significance is indicated with **P < .01 as compared to unstimulated cells. (b) Cells were preincubated with SB202190 (1 *μ*M) or BIRB 796 (100 nM) for 30 min, stimulated with cytokine mixture for 6 h, and harvested for total RNA extraction. The expression of iNOS mRNA was determined by quantitative real-time RT-PCR, and mRNA expression was normalized against GAPDH mRNA. Results are expressed as a percentage of CM, mean ± S.E.M., *n* = 6. One-way ANOVA with Bonferroni's posttest was performed, and statistical significance is indicated with ***P < .001 as compared to cells treated with CM.

The expression of iNOS mRNA over time was investigated in A549 cells. Cells were stimulated with the cytokine mixture for 0–6 h, and iNOS mRNA levels were measured. iNOS mRNA expression was increased in response to the stimulation with cytokine mixture up to 6 h (Figure 2(a)). The effect of p38 MAPK inhibitors on iNOS mRNA expression was investigated. Cells were preincubated with SB202190 (1 *μ*M) and BIRB 796 (100 nM) for 30 min and stimulated for 6 h. SB202190 and BIRB 796 inhibited the expression of iNOS mRNA in A549 cells (Figure 2(b)). 

**Figure 3:**
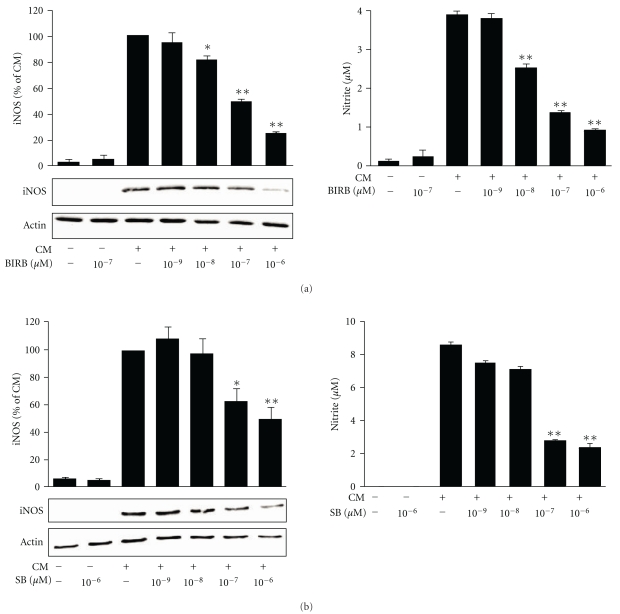
The effect of p38 MAPK inhibitors BIRB 796 and SB202190 on iNOS expression and NO production in response to cytokine mixture in A549 cells. A549 cells were preincubated with increasing concentrations of (a) BIRB 796 or (b) SB202190 for 30 min and stimulated with a cytokine mixture (CM: TNF, IFN*γ*, and IL-1*β*; 10 ng/mL each) for 24 h. iNOS expression was detected by Western blot. The gels are representatives of six separate experiments with similar results. Chemiluminescent signal was quantified, and iNOS (iNOS) expression was normalized to actin. NO production was measured as nitrite accumulated in the culture medium by Griess reaction. The results are expressed as mean ± S.E.M., *n* = 6. One-way ANOVA with Dunnett's posttest was performed, and statistical significance is indicated with *P < .05 and **P < .01 compared to cells treated with CM.

**Figure 4:**
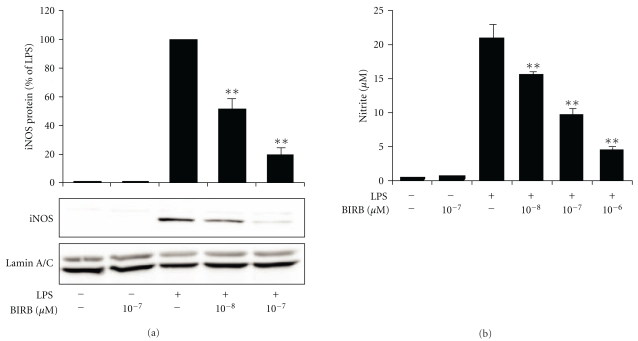
The effect of p38 MAPK inhibitor BIRB 796 on mouse iNOS protein expression and NO production in response to LPS in J774 cells. J774 cells were preincubated with increasing concentrations of BIRB 796 for 30 min and stimulated with LPS (10 ng/mL) for 24 h. iNOS protein expression was detected with Western blot. Chemiluminescent signal was quantified, and iNOS expression was normalized to lamin. The gels are representatives of six separate experiments with similar results. Results are expressed as a percentage of LPS, mean ± S.E.M., *n* = 6. NO production was measured as nitrite accumulated in the culture medium by Griess reaction, and the results are expressed as mean ± S.E.M., *n* = 6. One-way ANOVA with Dunnett's posttest was performed, and statistical significance is indicated with **P < .01 compared to LPS-treated cells.

The effects of p38 MAPK inhibitors SB202190 and BIRB 796 on iNOS protein expression and NO production were investigated. Cells were preincubated with SB202190 or BIRB 796 for 30 min and stimulated with the cytokine mixture (A549 cells) or LPS (J774 cells) for 24 h. Supernatants were collected and total cellular proteins were extracted for determination of nitrite production (a stable metabolite of NO in aqueous solution) and iNOS protein expression, respectively. Unstimulated cells did not express detectable levels of iNOS or produce NO, and pretreatment with SB202190 or BIRB 796 alone did not induce iNOS expression or NO production. Stimulation with the cytokine mixture or LPS induced iNOS protein expression and NO production, and SB202190 and BIRB 796 inhibited iNOS expression and NO production in A549 cells (Figure 3) and J774 cells (Figure 4) in a dose-dependent manner.

### 3.2. DUSP1 Negatively Regulated the Phosphorylation of p38 MAPK

**Figure 5:**
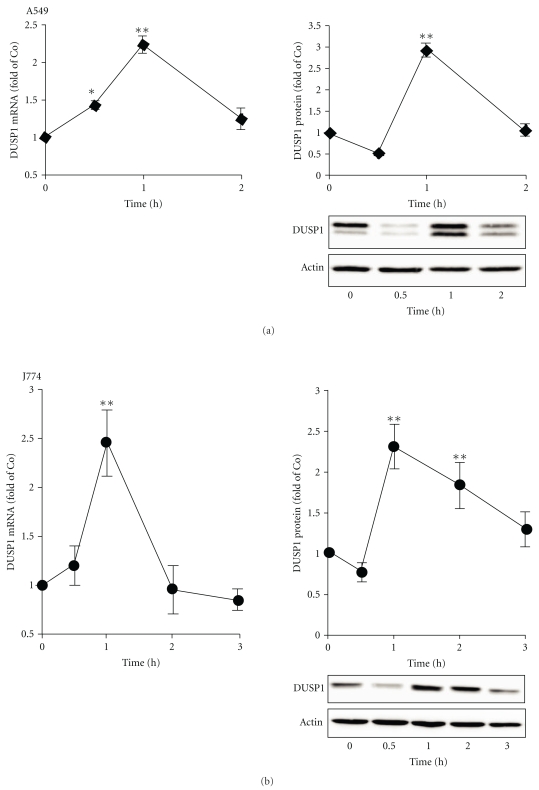
Expression of DUSP1 in response to cytokine mixture or LPS in A549 and J774 cells. Cells were stimulated with (a) cytokines (CM: TNF, IFN*γ*, and IL-1*β*; 10 ng/mL each) or (b) LPS (10 ng/mL) for the time indicated, and DUSP1 mRNA and protein expression was determined by quantitative real-time RT-PCR and Western blot, respectively. DUSP1 mRNA expression was normalized against GAPDH mRNA. In Western blots, chemiluminescent signal was quantified and DUSP1 protein expression was normalized against actin. The gels are representatives of six separate experiments with similar results. DUSP1 protein and mRNA levels are expressed in arbitrary units, DUSP1 expression in unstimulated cells (0 h) is set as 1, and the other values are related to that (mean ± S.E.M.; *n* = 6). One-way ANOVA with Dunnett's posttest was performed, and statistical significance is indicated with *P < .05 and **P < .01 compared with unstimulated cells.

**Figure 6:**
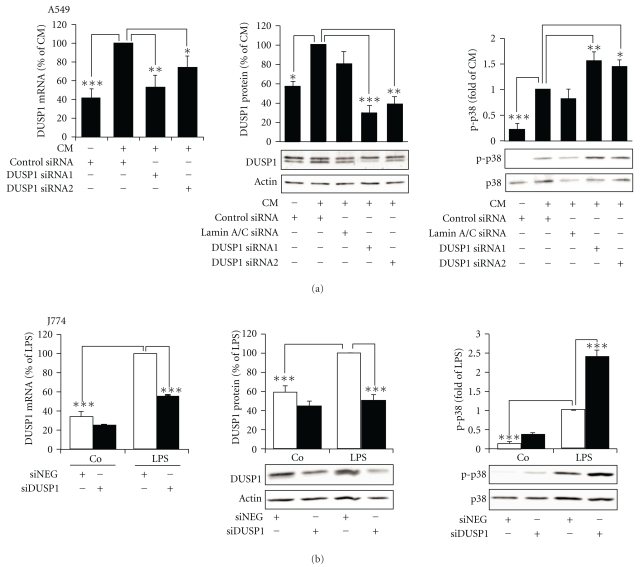
The effect of DUSP1 siRNA on DUSP1 expression and p38 MAPK phosphorylation in A549 and J774 cells. (a) A549 cells were transfected with DUSP1-specific siRNA 1 or 2 (DUSP1 siRNA 1 or 2), nontargeting control siRNA (control siRNA), or Lamin A/C-specific siRNA (Lamin A/C siRNA). (b) J774 cells were transfected with DUSP1-specific siRNA (siMKP-1) or nontargeting siRNA (siNEG). Cells were stimulated with cytokines (CM: TNF, IFN*γ*, and IL-1*β*; 10 ng/mL each) or LPS (10 ng/mL) for 1 h. DUSP1 mRNA expression was determined by quantitative real-time RT-PCR and normalized against GAPDH mRNA. DUSP1 protein expression and p38 MAPK phosphorylation were determined by Western blot. Chemiluminescent signal was quantified and DUSP1 expression was normalized against actin, and phosphorylated p38 MAPK was normalized against total p38 MAPK. Results are expressed as a percentage of CM or LPS. One-way ANOVA with Bonferroni's posttest was performed, and statistical significance is indicated with *P < .05, **P < .01, and ***P < .001, *n* = 6, except for DUSP1 mRNA in J774 cells, *n* = 3.

The expression of DUSP1 was investigated in A549 and J774 cells. A549 and J774 cells were stimulated with the cytokine mixture (TNF, IFN*γ*, and IL-1*β*) and LPS, respectively, and cells were then harvested for total RNA or protein extraction at the time points indicated. Unstimulated cells showed low-level basal DUSP1 protein (40 kDa) expression. DUSP1 mRNA and protein expression was enhanced by the cytokine mixture (A549 cells) or LPS (J774 cells). The maximal mRNA and protein expression was observed at 1 h after stimulation (Figure 5). DUSP1 mRNA expression was returned to basal level in 2 h in both cell types. DUSP1 protein expression was reduced near the basal level at 2 h and 3 h in A549 and J774 cells, respectively (Figure 5). In DUSP1 Western blots in A549 cells, an immunoreactive band of higher molecular weight was observed. The manufacturer's data sheet suggests this to be DUSP4. The immunoreactive band of higher molecular weight was not reduced by DUSP1 siRNA indicating that it is a molecule different from DUSP1 (Figure 6(a)). 

To investigate the effect of DUSP1 on the phosphorylation of p38 MAPK, we used siRNA to downregulate DUSP1 expression. In A549 cells transfected with two DUSP1-specific siRNAs, the protein and mRNA levels of DUSP1 were reduced as compared to the cells transfected with a nontargeting control siRNA showing that siRNA effectively downregulated DUSP1 (Figure 6(a)). The downregulation of DUSP1 by siRNA resulted in an increased p38 MAPK phosphorylation in response to stimulation at 1 h in A549 cells (Figure 6(a)). Lamin A/C siRNA, used as a positive control, downregulated lamin A/C mRNA by approximately 67% (*n* = 3, data not shown), but it did not affect DUSP1 expression or p38 MAPK phosphorylation in A549 cells (Figure 6(a)). The effect of DUSP1 siRNA on DUSP1 expression and p38 MAPK phosphorylation was investigated in J774 cells also. DUSP1 siRNA inhibited DUSP1 mRNA and protein expression and enhanced p38 MAPK phosphorylation in J774 cells (Figure 6(b)). These results show that DUSP1 catalyzed the dephosphorylation of p38 MAPK and thereby inactivated p38 MAPK in A549 and J774 cells.

### 3.3. DUSP1 Negatively Regulated iNOS Expression and NO Production

**Figure 7:**
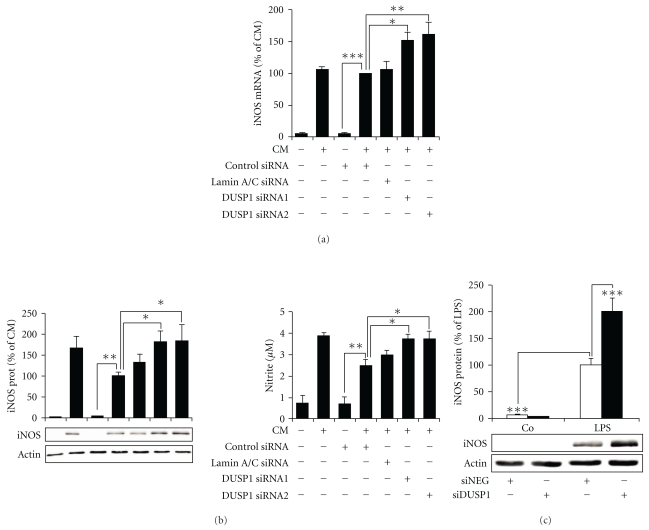
The effect of DUSP1 siRNA on iNOS mRNA and protein expression and NO production in A549 and J774 cells. A549 cells were transfected with DUSP1-specific siRNA 1 or 2 (DUSP1 siRNA 1 or 2), nontargeting control siRNA (control siRNA) or Lamin A/C-specific siRNA (Lamin A/C siRNA), and untransfected cells were used as controls. (a) Cells were stimulated with the cytokine mixture (CM: TNF, IFN*γ*, and IL-1*β*; 10 ng/mL each) for 6 h, and iNOS mRNA was determined with quantitative real-time PCR and normalized against GAPDH mRNA. (b) Cells were stimulated with CM for 24 h, and iNOS protein expression was detected by Western blot and NO production as nitrite by Griess reaction. (c) J774 cells were transfected either with DUSP1-specific siRNA (siDUSP1) or nontargeting siRNA (siNEG). Cells were stimulated with LPS (10 ng/mL) for 24 h, and iNOS protein expression was detected by Western blot. In Western blots, chemiluminescent signal was quantified and iNOS expression was normalized against actin. Results are expressed as related to cells treated with CM or LPS. One-way ANOVA with Bonferroni's posttest was performed, and statistical significance is indicated with *P < .05; **P < .01 and ***P < .001, *n* = 6.

The effect of down-regulation of DUSP1 on iNOS expression and NO production in response to the cytokine mixture (TNF, IFN*γ*, and IL-1*β*; A549 cells) or LPS (J774 cells) was investigated. A549 cells were transfected with two DUSP1-specific siRNA. Cells were then stimulated with the cytokine mixture for 6 h (iNOS mRNA analysis) and 24 h (iNOS protein analysis and NO production). Silencing of DUSP1 by siRNA resulted in increased iNOS mRNA and protein expression and NO production in A549 cells (Figures 7(a) and 7(b)). Human iNOS mRNA expression induced by cytokine mixture in untransfected cells was comparable to that seen in transfected cells (Figure 7(a)). Untransfected cells expressed human iNOS protein at somewhat higher level and produced slightly more NO as compared to the transfected cells (Figure 7(b)). Similarly, down-regulation of DUSP1 by siRNA increased iNOS protein expression in J774 cells (Figure 7(c)). In A549 cells, Lamin A/C-specific siRNA did not affect iNOS expression or NO production (Figures 7(a) and 7(b)) although it downregulated lamin A/C expression by about 70%. This strongly suggests that the increased iNOS expression and NO production in A549 cells caused by DUSP1-specific siRNA (Figures 7(a) and 7(b)) were due to the down-regulation of DUSP1 and not to nonspecific effects of siRNA, or general activation of RNA-induced silencing complex (RISC) pathway.

**Figure 8:**
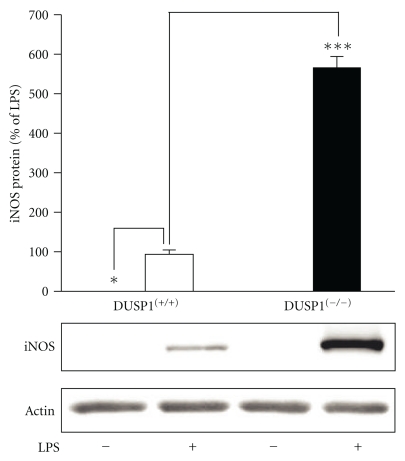
iNOS protein expression in bone marrow macrophages from wild-type and DUSP1-deficient mice. Bone marrow-derived macrophages differentiated from wild-type (DUSP1^+/+^ and DUSP1-deficient (DUSP1^−/−^) mice were incubated with LPS (10 ng/mL) for 24 h, and iNOS protein was measured by Western blot. The gels are representatives of three separate experiments with similar results. Chemiluminescent signal was quantified, and iNOS expression was normalized to actin. Results are expressed as a percentage of LPS, mean ± S.E.M. (*n* = 3). One-way ANOVA with Bonferroni's posttest was performed, and statistical significance is indicated with *P < .05 and ***P < .001.

To further confirm the effect of DUSP1 on iNOS expression, the induction of iNOS expression by LPS was investigated in bone marrow macrophages (BMMs) from DUSP1^(−/−)^ and wild-type mice. BMMs were stimulated with LPS (10 ng/mL) for 24 h. Cells were harvested for protein extraction, and iNOS expression was analyzed by Western blot. Unstimulated cells from wild-type and DUSP1^(−/−)^ mice did not express detectable amounts of iNOS. LPS enhanced iNOS expression in BMMs, and LPS-induced iNOS expression was markedly enhanced in BMMs isolated from DUSP1^(−/−)^ mice as compared to cells from wild-type mice (Figure 8).

## 4. Discussion

In the present study, we investigated the effect of DUSP1 on the expression of iNOS and production of NO in response to stimulation with cytokines (TNF, IFN*γ*, and IL-1*β*) in human A549 lung epithelial cells and with LPS in murine J774 macrophages and primary mouse BMMs. The main finding of this study was that DUSP1 negatively regulates iNOS expression and NO production by inhibiting the p38 MAPK phosphorylation both in mouse and human cells. This is the first study showing that DUSP1 regulates iNOS expression in human cells. 

Structurally distinct p38 MAPK inhibitors SB202190 and BIRB 796 have been reported to inhibit p38 MAPK at concentration range of 100 nM to 1 *μ*M in kinase assays [50]. We have previously reported that SB202190 and BIRB 796 inhibit phosphorylation of MK2 (a p38 MAPK substrate) in a dose-dependent manner with maximal inhibition at 1 *μ*M and 100 nM concentrations, respectively [47]. In the present study, SB202190 and BIRB 796 inhibited MK2 phosphorylation at these concentrations showing that both these inhibitors effectively inhibited p38 MAPK function in A549 and J774 cells. iNOS mRNA expression was reduced by both p38 MAPK inhibitors at these concentrations in A549 cells. Also, iNOS protein expression and NO production were reduced by SB202190 and BIRB 796 in a dose-dependent manner in both A549 and J774 cells. iNOS expression has been reported to be inhibited by a p38 MAPK inhibitor SB203580, a compound structurally related to SB202190, at corresponding concentrations in human cells [13], and by SB203580 and SB202190 in J774 cells [35]. 

DUSPs are protein phosphatases capable to dephosphorylate tyrosine and threonine/serine residues and, hence, regulate the activity of their target proteins. A subgroup of DUSPs target MAPKs and dephosphorylate tyrosine and threonine residues in MAPKs. Currently, at least ten MAPK-associated DUSPs have been identified and they differ from each other by substrate specificity, tissue distribution, cellular localization, and expressional pattern [40, 44]. DUSP1 is a nuclear phosphatase inducible by LPS and cytokines, and it has substrate specificity towards p38 MAPK and JNK. DUSP1 has been shown to regulate the phosphorylation of p38 MAPK and JNK in primary mouse macrophages [42, 43, 51] and endothelial cells [52]. We have recently reported that DUSP1 regulates the phosphorylation of both p38 MAPK and JNK in A549 human pulmonary epithelial cells [47] and p38 MAPK in chondrocytes [48]. In the present study, stimulation with cytokines or LPS enhanced the expression of DUSP1 in A549 and J774 cells. Transfection of DUSP1-specific siRNA decreased DUSP1 protein and mRNA expression and resulted in enhanced p38 MAPK phosphorylation, iNOS expression, and NO production in A549 cells. This is the first report showing that DUSP1 regulates human iNOS expression. 

Down-regulation of DUSP1 by siRNA increased iNOS expression also in murine macrophages. Accordingly, LPS-induced iNOS expression was enhanced in BMMs isolated from DUSP1-deficient mice, which confirmed our results with cells in which DUSP1 had been silenced with siRNA. These results are also in line with the previous reports showing that iNOS expression is increased in DUSP1-deficient mice in response to low-dose LPS administration or septicemia due to Gram-positive bacteria *in vivo* [45, 46]. Interestingly, *E. coli* infection in DUSP1-deficient mice was reported to result in reduced serum nitrate levels and lower iNOS expression in liver as compared to wild-type mice [53]. These conflicting findings may reflect the differences in innate immune response to purified cell wall component (LPS) of Gram-negative bacteria and viable bacteria.

Translation of the results describing the mechanisms of mouse iNOS expression to that of human is not always straightforward. The size of the iNOS promoter differs greatly between mouse (~1.5 kb) and human (up to 16 kb). LPS and IFN*γ* induce mouse iNOS promoter activity 50–100-fold [5]. Human iNOS promoter shows low basal activity in the absence of measurable mRNA [19, 54]. Cytokine stimulation increases human iNOS promoter activity only approximately 7-to-10 fold, while mRNA expression increases more than 100-fold [8, 19]. This demonstrates the differences in transcriptional but also in posttranscriptional regulation of iNOS expression between mouse and human cells. From a therapeutic point of view, the mechanisms regulating iNOS expression ought to be applicable for human iNOS expression. In this study, we demonstrated the negative regulation of human iNOS expression by DUSP1, which has not previously been reported. Also, DUSP1 regulated mouse iNOS expression. According to our results, p38 MAPK/DUSP1 pathway seems to be a conserved signaling/regulatory mechanism for iNOS expression across species despite the considerable other differences in the regulation of iNOS expression between mouse and human. p38 MAPK/DUSP1 pathway may regulate iNOS expression both at transcriptional and posttranscriptional levels. Human iNOS has been shown to be regulated by p38 MAPK by a mechanism dependent on the interaction between human iNOS mRNA 3′ untranslated region and tristetraprolin, an mRNA-binding protein. p38 MAPK positively regulates tristetraprolin, which stabilizes iNOS mRNA and leads to increased iNOS expression [13]. In addition, human and rodent iNOS promoter activity has also been reported to be regulated by p38 MAPK [11, 55, 56]. 

Therapeutic approaches targeted to enhance DUSP1 expression or its function may provide a novel mechanism for anti-inflammatory treatment. Glucocorticoids enhance DUSP1 expression, and some of the anti-inflammatory effects of glucocorticoids, such as the inhibition of cytokine and chemokine expression, are mediated by DUSP1 [57, 58]. In alveolar macrophages from patients with glucocorticoid-resistant asthma, the expression of DUSP1 in response to glucocorticoids was reported to be reduced and, correspondingly, p38 MAPK phosphorylation was increased [59]. Also, disease-modifying antirheumatic drug aurothiomalate was recently found to enhance DUSP1 expression in chondrocytes and cartilage, and DUSP1 mediated its inhibitory effect on COX-2, IL-6, and matrix metalloproteinase 3 expression [48]. Interestingly, DUSP4, another MAPK-associated DUSP, has been reported to regulate inflammatory response via ERK and DUSP1. DUSP4-deficient mice were protected from the excessive inflammatory response during septic infection. These animals showed increased ERK phosphorylation due to DUSP4 deficiency. Increased ERK activity resulted in enhanced DUSP1 expression and, in turn, reduced TNF and IL-6 production by macrophages [60]. 

In addition to inflammatory conditions, increased iNOS-derived NO production has been shown to be present in various solid tumors. Myeloid-derived suppressor cells (MDSCs) are a group of myeloid progenitor cells, immature macrophages, granulocytes, and dendritic cells capable of suppressing functions of T cells. In malignancies, they infiltrate in solid tumors and promote tumor growth. Tumor-associated monocytic MDSCs express iNOS at high level. iNOS-derived NO by MDSCs targets tumor infiltrating T cells and suppresses their functions by inhibiting T cell receptor signaling and Jak/STAT pathway activation and inducing T cell apoptosis. NO production is one of the central mechanisms by which MDSCs promote tumor growth [61]. IL-6 and granulocyte/monocyte colony-stimulating factor (GM-CSF) have been reported to induce the differentiation of MDSCs from peripheral blood mononuclear cells [62]. Interestingly, DUSP1 suppresses IL-6 and GM-CSF, expression [41, 47, 63]. DUSP1 may limit the differentiation and functions on MDSCs by suppressing the expression of IL-6, GM-CSF and iNOS. DUSP1 has been shown to be upregulated in early phases of epithelial carcinogenesis in bladder, colon, and prostate cancers with progressive loss on expression with higher histological grades and in metastasis [64]. In lung cancer, DUSP1 predicted improved survival [65]. *In vitro*, DUSP1 overexpression induced apoptosis at colon cancer cells [66]. These findings imply that DUSP1 may have antitumor activity. However, the antitumor effects of DUSP1 may well be related to the cancer type and the stage of the disease. It is noteworthy that DUSP1 has been recently linked to the depressive behavior in animal experiments [67], and if this appears to be the case also in humans, it may limit the therapeutic potential of DUSP1 in inflammatory and other conditions.

## 5. Conclusions

In conclusion, our results show that DUSP1 negatively regulated iNOS expression and NO production induced by inflammatory stimuli by inhibiting p38 MAPK phosphorylation in murine and human cells. This study extends our understanding on the role of DUSP1 in inflammation and on the mechanisms that regulate iNOS expression especially in human cells. This may give new insights in the development of novel drug treatments for diseases complicated with increased iNOS-mediated NO production.

## Figures and Tables

**Table 1:** Primer and probe sequences for quantitative RT-PCR.

Gene	Oligonucleotide	Sequence	Conc. (nM)
Human GAPDH	Forward primer	TCCTACCACCAGCAACCCTGCCA	300
	Reverse primer	GCAACAATATCCACTTTACCAGAGTTAA	300
	Probe	CGCCTGGTCACCAGGGCTGC	150
Human iNOS	Forward primer	GCAGGTCGACTATTTCTTTCA	300
	Reverse primer	TCCTCCTCCGCCTCGTAAGGA	300
	Probe	TCAAGAGCCAGAAGCGCTATCACGAAGATA	150
Mouse GAPDH	Forward primer	GCATGGCCTTCCGTGTTC	300
	Reverse primer	GATGTCATCATACTTGGCAGGTTT	300
	Probe	TCGTGGATCTGACGTGCCGCC	150
Mouse iNOS	Forward primer	CCTGGTACGGGCATTGCT	300
	Reverse primer	GCTCATGCGGCCTCCTT	300
	Probe	CAGCAGCGGCTCCATGACTCCC	150
Mouse DUSP1	Forward primer	CTCCTGGTTCAACGAGGCTATT	300
	Reverse primer	TGCCGGCCTGGCAAT	300
	Probe	CCATCAAGGATGCTGGAGGGAGAGTGTT	150

## Abbreviations

AP-1: Activating protein-1
BMMs: bone marrow macrophages
COX-2: Cyclooxygenase-2
DUSP: Dual specificity phosphatase
ERK: Extracellular signal-regulated kinase
GAPDH: Glyceraldehyde-3-phosphate dehydrogenase
GM-CSF: Granulocyte/monocyte colony-stimulating factor
iNOS: Inducible nitric oxide synthase
IFN*γ*: Interferon-*γ*
IRF: Interferon-regulatory factor
IL: Interleukin
JNK: Jun N-terminal kinase
LPS: Lipopolysaccharide
M-CSF: Macrophage colony-stimulating factor
miRNA: MicroRNA
MK2: Mitogen-activated protein kinase-activated protein kinase 2
MAPK: Mitogen-activated protein kinase
MDSC: Myeloid-derived suppressor cell
NO: Nitric oxide
NOS: Nitric oxide synthase
Oct-1: Octamer factor
siRNA: Small interfering RNA
TNF: Tumor necrosis factor.
